# Impacts of Ocean Acidification on Early Life-History Stages and Settlement of the Coral-Eating Sea Star *Acanthaster planci*

**DOI:** 10.1371/journal.pone.0082938

**Published:** 2013-12-16

**Authors:** Sven Uthicke, Danilo Pecorino, Rebecca Albright, Andrew Peter Negri, Neal Cantin, Michelle Liddy, Symon Dworjanyn, Pamela Kamya, Maria Byrne, Miles Lamare

**Affiliations:** 1 Australian Institute of Marine Science, Townsville, Queensland, Australia; 2 Department of Marine Biology, University of Otago, Dunedin, Otago, New Zealand; 3 National Marine Science Centre, Southern Cross University, Coffs Harbour, New South Wales, Australia; 4 Schools of Medical and Biological Sciences, University of Sydney, Sydney, New South Wales, Australia; University of New South Wales, Australia

## Abstract

Coral reefs are marine biodiversity hotspots, but their existence is threatened by global change and local pressures such as land-runoff and overfishing. Population explosions of coral-eating crown of thorns sea stars (COTS) are a major contributor to recent decline in coral cover on the Great Barrier Reef. Here, we investigate how projected near-future ocean acidification (OA) conditions can affect early life history stages of COTS, by investigating important milestones including sperm motility, fertilisation rates, and larval development and settlement. OA (increased *p*CO_2_ to 900–1200 µatm *p*CO_2_) significantly reduced sperm motility and, to a lesser extent, velocity, which strongly reduced fertilization rates at environmentally relevant sperm concentrations. Normal development of 10 d old larvae was significantly lower under elevated *p*CO_2_ but larval size was not significantly different between treatments. Settlement of COTS larvae was significantly reduced on crustose coralline algae (known settlement inducers of COTS) that had been exposed to OA conditions for 85 d prior to settlement assays. Effect size analyses illustrated that reduced settlement may be the largest bottleneck for overall juvenile production. Results indicate that reductions in fertilisation and settlement success alone would reduce COTS population replenishment by over 50%. However, it is unlikely that this effect is sufficient to provide respite for corals from other negative anthropogenic impacts and direct stress from OA and warming on corals.

## Introduction

Carbon dioxide (CO_2_) concentrations in the atmosphere have increased by 40% over the past 250 years and are likely to double by the end of this century [1]. Increased atmospheric CO_2_ leads to increased sea surface temperatures and a reduction in ocean pH, decreased carbonate and increased dissolved inorganic carbon (DIC) concentrations [2]. While some marine primary producers such as seagrasses, phytoplankton and macroalgae may benefit from increased DIC [3], [4], many calcifying organisms exhibit reduced calcification due to lower saturation states of carbonate (e.g. [5]–[7]). Coral reefs in particular are threatened both by increased temperature (more frequent coral bleaching events) and ocean acidification (OA) [8]. Although susceptibility of corals to increased *p*CO_2_ varies with species [9], it appears that many structurally complex corals will be lost, leading to a decline in habitat available to a variety of other species and changes to ecosystem structure and function [10].

In addition to the global pressures OA and climate warming, corals are under simultaneous threat from a variety of local stressors such as nutrient runoff, overfishing and cyclones that also dramatically shape the health of the system [11], [12]. Episodic outbreaks of the corallivorous crown of thorns seastars (COTS, *Acanthaster planci*) reduce coral cover on many reefs in the Indo-Pacific region [13] and are a major contributor to recent coral decline in the Great Barrier Reef (GBR) [12]. The underlying causes of these outbreaks have long been debated [13]; however, higher larval survivorship caused by greater food (phytoplankton) availability, driven by agricultural land runoff, is currently the most widely accepted hypothesis to explain primary COTS outbreaks [14].

Sexual reproduction and early life history stages of marine invertebrates can be highly vulnerable to OA [15], [16], with fertilization affected in some species but not others [17]–[20]. Calcifying larvae of echinoids (sea urchins) are vulnerable to reduced pH/increased *p*CO_2_ with reduced calcification, abnormal growth and lower survival rates [15], [21], [22]. Similar results have also been reported for non-calcifying asteroid (sea star) larvae [17], [23]. Reductions in settlement success due to OA have not been investigated in echinoderms, but have been documented in corals [24]–[27]. Here, we examine the effects of OA on sperm motility, fertilisation, larval development and larval settlement in the corralivorous sea star *Acanthaster planci*. This is the first study to comprehensively investigate several of these life history stages and also include settlement for an important marine species, and to provide a minimum estimate of the effects on population replenishment.

## Results

**Figure 1:**
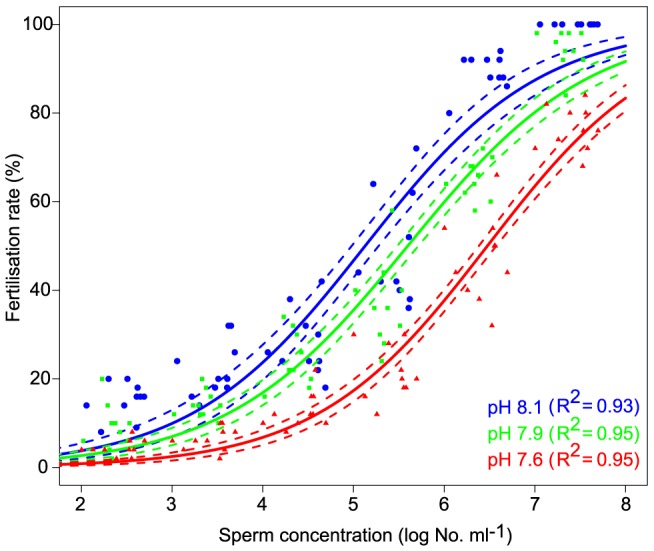
Fertilisation success of *A. planci* oocytes across a range of sperm concentrations (10^2^–10^8^ sperm ml^−1^) under three pH/*p*CO_2_ conditions. Solid lines represent best-fit curves under three different pH/*p*CO_2_ scenarios, and corresponding dashed lines are 95% confidence intervals.

**Figure 2:**
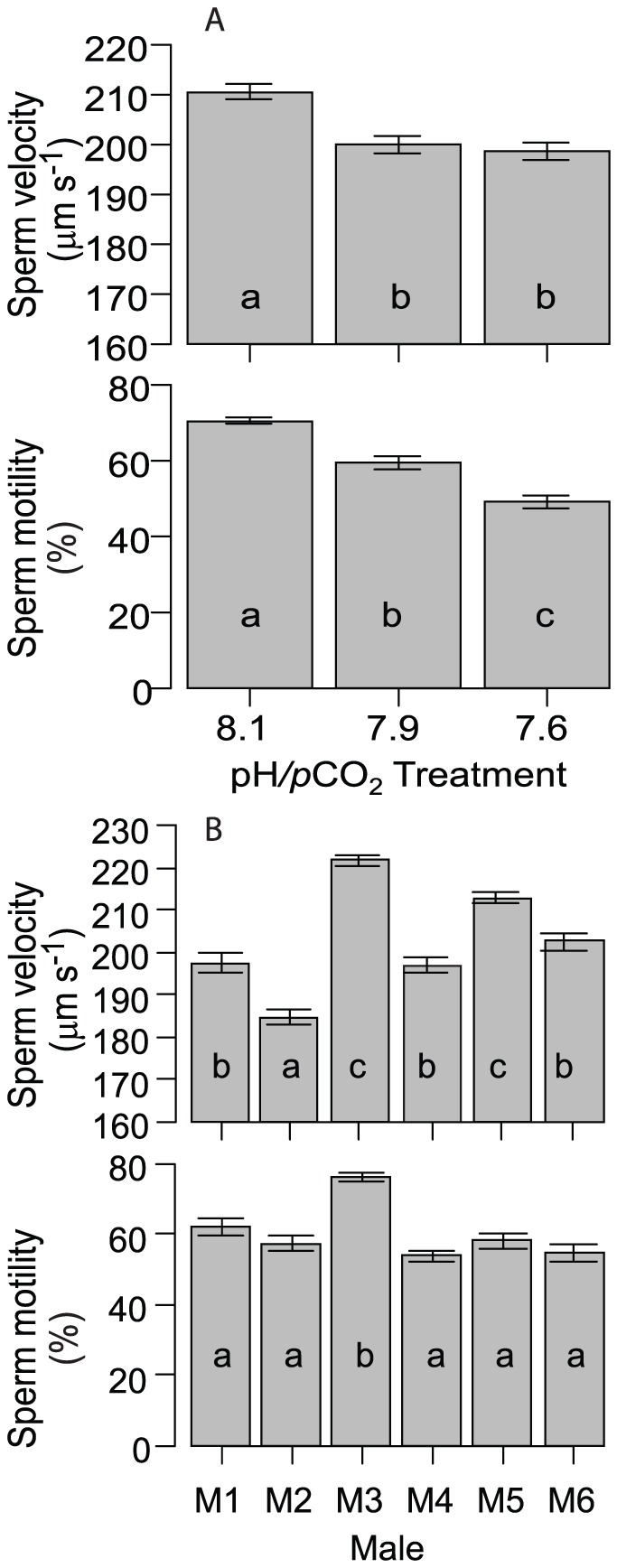
A) Sperm velocity and percentage of motile sperm (% motility) for the three different treatments (Factor Treatment, see ANOVA in Table 3). B) Differences in sperm velocity and percentage of motile sperm between individual males (Factor Male, see ANOVA in Table 3. Error bars represent 1 standard error. Averages with different letters are significantly different (p<0.05, Tukey-Kramer posthoc tests).

Fertilisation rates depended on sperm concentrations under three different pH/*p*CO_2_ conditions (Figure 1, see Table 1 for carbon chemistry parameters for all experiments). Functions for the three pH/*p*CO2 conditions tested had a similar slope (Table 2), but inflection points (ie the sperm concentration at which 50% fertilisation occurs) were significantly different among treatments, and increased with increasing *p*CO_2_ (Table 2, ‘right-shift’ in Figure 1). The percentage of fertilisation was reduced by >7% at pH_NBS_ 7.9 (*p*CO_2_: 877 µatm) and 25% at pH_NBS_ 7.7 (*p*CO_2_: 1658 µatm) across sperm concentrations spanning more than 3 orders of magnitude (∼10^4^ to 10^7^ sperm ml^−1^). This reduction in fertilisation success is coincident with a significant reduction in sperm velocity and the percentage of motile (i.e. moving) sperm at elevated *p*CO_2_, with significant variation among individual males (Table 3, Figure 2). The coelomic fluid surrounding gonads of *A. planci* had a mean pH_NBS_ of ∼7.49 (N = 5, 95% CI: 7.39–7.61).

**Table 1:** Seawater chemistry for different experiments on *Acanthaster planci*.

Experiment/Nominal Target pH_NBS_		N	pH _[NBS/derived]_	A_T_ [µmol/kg]	DIC [µmol/kg]	*p*CO_2_ [µatm]	Ω_Ca_	Ω_Ar_
Fertilisation:	8.1	1	8.11	2349	2071	520	4.89	3.25
	7.9	1	7.91	2353	2174	877	3.40	2.26
	7.6	1	7.67	2356	2278	1658	2.06	1.37
Sperm:	8.1	2	8.11 (8.11–8.11)	2333 (1)	2050 (0)	499 (7)	4.96 (0.02)	3.03 (0.01)
	7.9	2	7.84 (7.84–7.84)	2337 (1)	2190 (0)	1053 (7)	2.92 (0.02)	1.94 (0.01)
	7.6	2	7.61 (7.61–7.61)	2339 (1)	2281 (2)	1892 (26)	1.83 (0.02)	1.21 (0.01)
Larvae:	8.1	10	8.09 (8.05–8.12)	2329 (15)	2062 (21)	536 (60)	4.68 (0.34)	3.11 (0.22)
	7.8	10	7.78 (7.76–7.80)	2329 (15)	2207 (17)	1211 (36)	4.02 (1.02)	2.67 (0.68)
	7.6	10	7.57 (7.55–7.59)	2329 (15)	2284 (14)	2056 (12)	3.36 (1.02)	2.24 (0.68)
CCA: Pre-Ind.	8.2	7	8.23 (8.18–8.29)	2337 (16)	1995 (27)	363 (56)	5.85 (0.62)	3.88 (0.42)
Present	8.1	7	8.15 (8.13–8.16)	2337 (10)	2047 (14)	458 (18)	5.03 (0.22)	3.33 (0.16)
	8.0	7	8.05 (8.04–8.11)	2342 (12)	2100 (28)	598 (84)	4.25 (0.25)	2.81 (0.16)
	7.9	7	7.94 (7.89–8.00)	2339 (12)	2158 (22)	806 (111)	3.44 (0.24)	2.28 (0.16)
Settlement: Pre-Ind.	8.2	1	8.25	2347	1979	341	6.31	4.20
Present	8.1	1	8.14	2345	2047	471	5.19	3.46
	8.0	1	8.04	2350	2104	609	4.40	2.93
	7.9	1	7.95	2350	2151	783	3.69	2.45

Nominal target pH represents the pH value used to denominate treatments throughout the text. pH_derived_ is accurate pH value as derived from DIC and AT measurements; all pH values are given on the NBS scale. Upper and lower 95% confidence intervals for pH and standard deviations for all other parameters are shown in parentheses. *p*CO_2_, calcite saturation state (Ω_Ca_) and aragonite saturation state (Ω_Ar_) were calculated based on measured total alkalinity (A_T_) and dissolved inorganic carbon (DIC). N =  number of samples. Pre-Ind.: Pre-Industrial treatment. Sperm: experiments on sperm velocity and percent of motile sperm; larvae: experiment on larval development, CCA: conditions during crustose coralline algal culture.

**Table 2:** Results of the logistic regression for fertilisation rates at three different pH/*p*CO_2_ conditions, all curve fits were highly significant (p<0.0001).

	Estimate (SE)	T	P	R^2^
8.1				
b	1.04 (0.07)	14.4	<0.0001	0.93
c	5.13 (0.07)	69.51	<0.0001	
7.9				
b	1.00 (0.05)	18.25	<0.0001	0.95
c	5.60 (0.06)	96.64	<0.0001	
7.6				
b	1.06 (0.05)	19.38	<0.0001	0.95
c	6.45 (0.05)	135.23	<0.0001	

b =  slope of the curves; c =  x-value at the inflection point of the curve, i.e. the sperm concentration at which 50% of the eggs were fertilised.

**Table 3:** Results of mixed model analysis of variance for sperm velocity and percentage of motile sperm (% motile) of *A. planci* sperm subjected to three different pH/*p*CO_2_ conditions (factor pH).

			Velocity			% Motile	
	DF	MS	F	p	MS	F	p
pH	2	0.0218	23.57	<0.0001	1.2600	23.86	<0.0001
Male	5	0.0381	41.23	<0.0001	0.4400	8.33	<0.0001
pH X M	10	0.0015	1.58	0.1558	0.0531	1.01	0.4588
Slide (pH X M)	34	0.0009	1.99	<0.0001	0.0528	8.32	<0.0001
Residual	232	0.0005			0.0063		
Total	283						

Sperm was derived from 6 separate males (Males). Slide is a random/nested factor representing measurements taken from three independent dilutions for each male x pH combination. Velocity data were log-transformed, motility data arcsine-square root-transformed for analysis.

**Figure 3:**
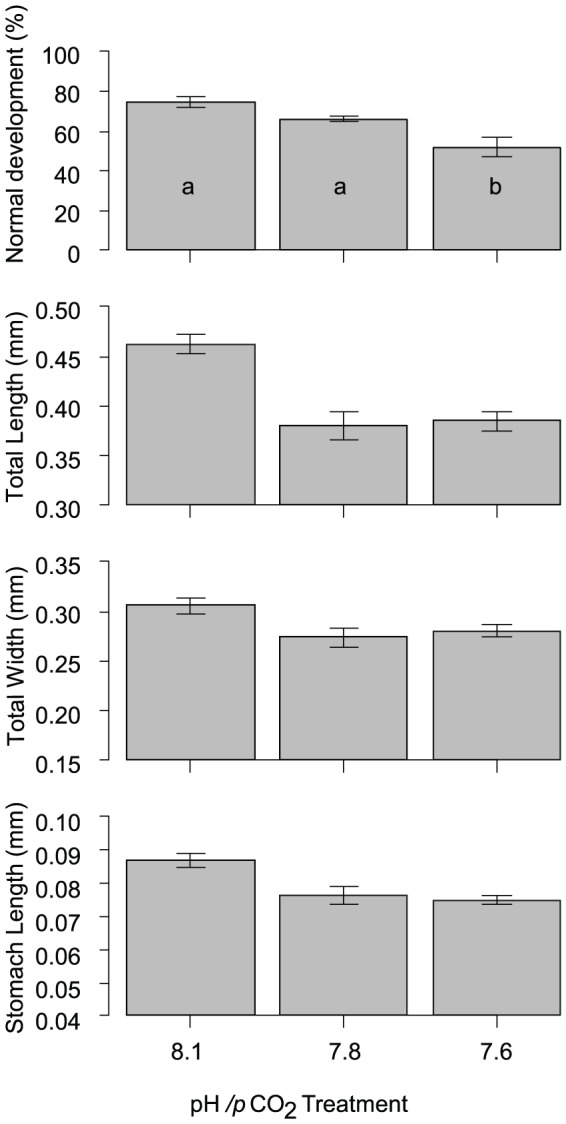
Percentage of normal development and several morphometric parameters for *Acanthaster planci* larvae grown for 10 d under control and two reduced pH/increased *p*CO_2_ conditions. Error bars represent 1 standard error. Averages with different letters are significantly different (p<0.05, Tukey-Kramer posthoc tests).

By 10 d, the majority of larvae in all treatments had reached late bipinnaria to early brachiolaria stages. The percentage of normal developing larvae declined by ∼13% at pH_NBS_ 7.6 compared with controls (Figure 3) and this difference was significant (ANOVA, F_2,12_ = 12.34, p = 0.0012; Tukey-Kramer posthoc test, p<0.05). Larval morphometry (e.g., length, stomach width) was not significantly different between treatments (Figure 3; Table 4).

**Table 4:** Results of mixed model analysis of variance for morphometric parameters of *A. planci* larvae cultured for 10 d in three different pH/*p*CO_2_ conditions (factor pH).

		Larval length			Larval Width			Stomach length		
	DF	MS	F	p	MS	F	p	MS	F	p
pH	2	0.0107	4.02	0.0567	0.0016	0.60	0.5677	0.00041	3.14	0.0924
Container (T)	9	0.0027	6.21	<0.0001	0.0026	12.09	<0.0001	0.00013	5.69	<0.0001
S	145	0.0004			0.0002			0.00002		
Total	156									

Container is a random/nested factor representing measurements taken from four independent culture containers. All data were log-transformed prior to analysis.

By 17 d, larvae were advanced brachiolaria stage, and a high proportion (75.4%) had a well-developed rudiment and were competent to settle. Two settlement experiments were conducted in parallel, achieving typically [28] moderate levels of settlement success of 10–20% in ambient conditions. In the first experiment settlement substrata known to induce settlement in COTS (CCA -crustose coralline red algae, and/or biofilms) that had developed under four pH conditions for 85 d were used. In the second experiment CCA was only exposed to ambient conditions prior to the settlement tests which were then conducted under the four different pH/*p*CO_2_ conditions.

**Figure 4:**
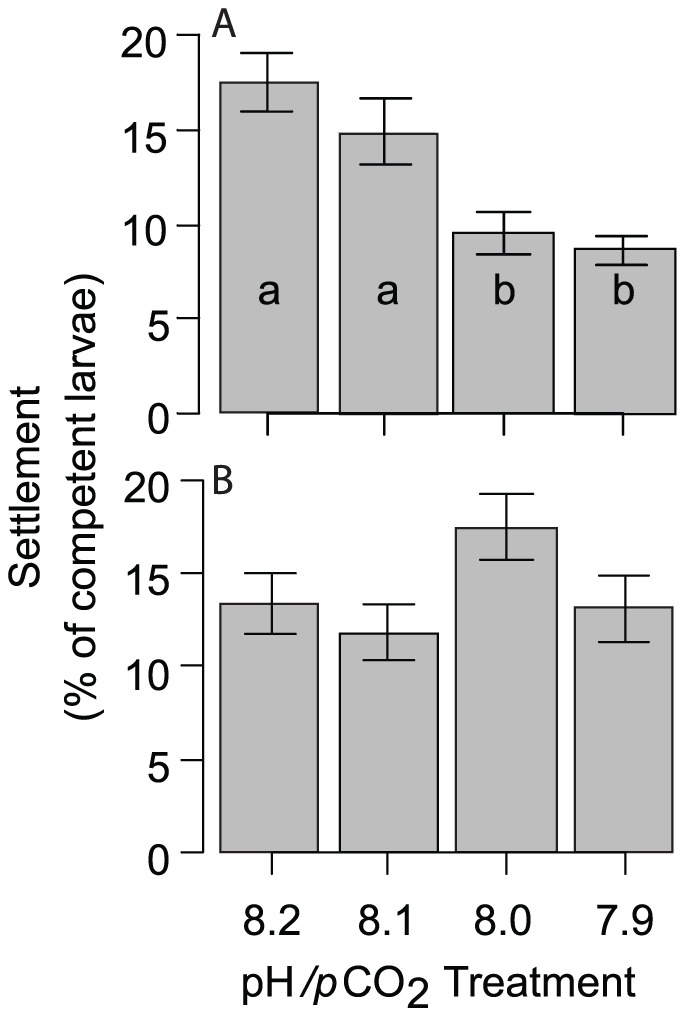
A) Settlement success of *A. planci* larvae on CCA and biofilms exposed for 85 d in water with pre-industrial, present day control or two near future CO_2_ conditions. B) Control experiment with CCA all pre-incubated under ambient conditions, and only the experimental water differing in pH/*p*CO_2_. The pH values given on the x-axis represent target values on the NBS scale (see Table 1). Error bars represent standard errors, means with the same index letters are not significantly different (Tukey-Kramer posthoc, test, p>0.05). A

There was no difference in the settlement success between CCA and biofilms (Table 5). In contrast, differences between pH/*p*CO_2_ treatments were significant, with settlement on pre-exposed CCA and biofilms declining by about 50% from the pre-industrial treatment (pH_NBS_ 8.1, *p*CO_2_ = 350 µatm) to the elevated *p*CO_2_ (pH_NBS_ 7.8, *p*CO_2_ = 800 µatm) treatment (Figure 4). Settlement success for larvae offered CCA cultured under present day *p*CO_2_ conditions but in seawater of the four pH levels was not significantly different (ANOVA, Figure 4); indicating that changes in settlement response was not due to the seawater pH/*p*CO_2_ directly.

**Table 5:** Analysis of variance (type III sums of squares) for settlement of COTS larvae on CCA and biofilms exposed for 85 d in water with pre-industrial, present day control or two near future *p*CO_2_ conditions (see Table 1).

	Df	MS	F	P
pH/*p*CO_2_	3	0.0806	11.42	<0.0001
CCA vs Biofilm	1	0.0012	0.17	0.6821
Interaction	3	0.0053	0.76	0.5222
Residuals	67	0.0071		

Data were arcsine-square root-transformed for analysis.

**Figure 5:**
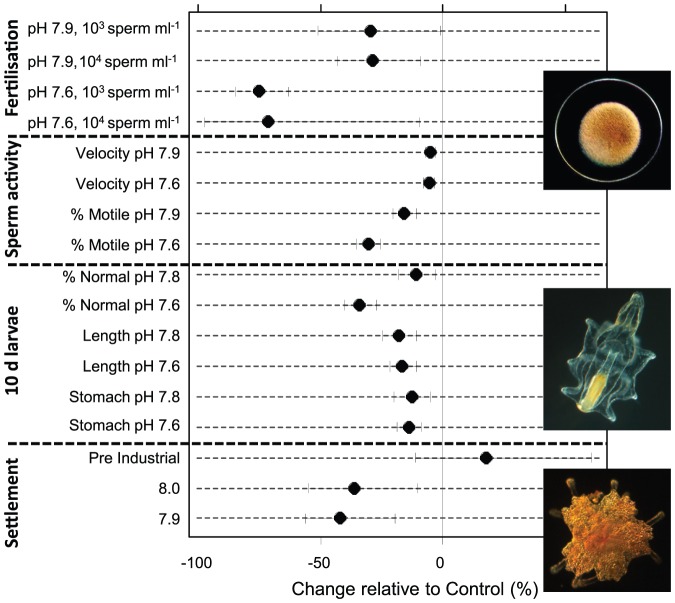
Average effect size (expressed as % change from present day conditions) control and 95% Bayesian confidence intervals for all early live history parameters which were at least marginally (p<0.10) significant in frequentist analysis.

To evaluate the importance of individual early life history parameters for overall recruitment success, we calculated effect sizes and Bayesian confidence intervals for each parameter (Figure 5). Environmentally relevant sperm concentrations were estimated using a sperm diffusion model and parameters given in Babcock et al. [29]. Diffusion model results indicated that eggs released from females 0.5–10 m downstream of 4 spawning males experience sperm concentrations in the range of 10^3^–10^4^ ml^-1^. Under these conditions, fertilisation at pH_NBS_ 7.9 and 7.6 was reduced by 29% and 75%, respectively (Figure 5). Comparison of the effect sizes illustrated that the effect of pH/*p*CO_2_ on fertilisation was amongst the most distinct of all parameters measured. While both the reduction in the percentage of motile sperm and sperm swimming speed influence fertilization, effect sizes suggest that motility (16 and 30% reduction) may be the driving force behind reduced fertilization success at low pH. Effects on sperm velocity were comparatively small (5 and 6%). OA effects on larval development and growth were subtle (11–18% changes in the pH_NBS_ 7.8 treatment) and it is uncertain if these effects are of sufficient importance for overall development to affect recruitment rates and population maintenance. However, slower development will lead to an extended period in the plankton, and daily mortality rates for echinoderm larvae due to predation and other factors can be 5–15% [30]. With an average reduction of settlement on biofilms and CCA of 36%, effects on settlement were roughly in the same range as for fertilisation. Even if only the effects of pH/pCO_2_ on fertilisation and settlement are taken into account, overall recruitment under near future OA conditions may be reduced by ≈50% (100−(100*(1−0.29)*(1−0.36)) = 54.5%).

## Discussion

Near-future OA has the potential to severely impact population maintenance and growth of COTS by affecting several early life history stages including fertilisation and recruitment success. In the present study, depressed pH/elevated *p*CO_2_ affected the percentage of motile sperm more distinctly than velocity, similar to previous experiments on echinoids [19], [20]. Early work by Mortensen [31] already described that asteroid sperm requires pH elevation for activation. It is thought that decreased pH in the gonad suppresses activation of sperm mitochondria in echinoderms. Thus, sperm require elevated pH, such as that of the surrounding seawater, for activation of mitochondrial processes [18]. For example, the gonad of the sea star *Patiria pectinifera* has a pH of 6.4, and elevation of pH activates sperm [32]. Similarly, in the present study, the coelomic fluid of *A. planci* had a pH_NBS_ of ∼7.5, indicating sperm is also stored inactive at depressed pH. Previous studies on echinoids also show reductions the percentage of motile sperm at decreased pH and ultimately reproductive success [18]–[20]. A second mechanism for reduced fertilisation success at depressed pH involves reduced efficiency of the fast sperm block for polyspermia [18], although this would be evident as a drop off in fertilisation at higher sperm concentrations, an outcome we did not observe under the sperm concentration tested. Significant variation in sperm velocity and motility amongst males was also observed in sea urchins [20], and may provide a mechanism for future selection and potential adaptation of the species to increased *p*CO_2_. Recent population genomic [33] and quantitative genetic [34]–[36] studies on sea urchins suggest a potential for adaptation to OA through selection in that class of the Echinoderms. Whether this is also the case for COTS should be subject to further investigation.

Ocean acidification significantly reduces the growth of calcifying echinoderm larvae (e.g. [15], [21], [22], [37], [38]). Interestingly, non-calcifying asteroid larvae, such as *A. planci*, are also sensitive to OA; for example, increased mortality and a stunted growth were recently reported in a temperate and an Antarctic asteroid species [17], [23]. The underlying mechanism(s) responsible for OA-induced mortality and/or depressed growth of non-calcifying larvae is unclear, but it is possible that direct teratogenic effects of decreased pH stunted growth or that increased metabolism associated with acid/base regulation reduces energy availability, or ‘scope’ for growth [39] [40].

The present study is the first to experimentally demonstrate effects of OA on settlement of invertebrates other than corals. Both temperature and OA can alter biofilm and/or crustose coralline algal (CCA) communities that are critical for inducing settlement of coral larvae [24], [26], [27], [41]. Microbial studies demonstrated that OA alters the chemistry and bacterial community composition of settlement substrata [24], [42], but it is unclear which factor is ultimately responsible for reductions in larval settlement. Larval settlement success observed here on CCA not exposed to decreased pH was in the same range as those measured previously on the same algae [28]. That earlier study also demonstrated that addition of antibiotics reduced settlement, suggesting bacterial biofilms as possible agents of settlement induction. This is consistent with our observation that settlement success was similar on CCA and biofilms. Our second settlement experiment illustrated that reduced larval settlement under OA conditions (depressed pH/elevated *p*CO_2_) is not simply the direct result of sea water chemistry. These findings are consistent with those for coral larvae [24], [27]. Detecting similar trends in larval settlement in representatives from different tropical phyla, and given similar cues are important for the recruitment of urchin [43] and abalone [44] larvae, reduced settlement through OA altering settlement cues could be an ecologically important phenomenon also impacting polar and temperate species.

It is intriguing that projected ocean acidification scenarios have similar effects on early life history stages of prey (coral) and predator (COTS), despite representing widely divergent phylogenetic groups. Fertilisation curves derived here for COTS are similar to those observed for corals [26], [45], and settlement experiments yield similar results (see above). Our model calculations suggests that population replenishment of COTS could be reduced by at least 50% if *p*CO_2_ increases as expected by 2100. This estimate may be conservative, as it does not include additional unknown impacts of OA on juveniles and adults. However, the estimate also assumes populations are recruitment-limited as assumed for most open marine populations [46]. In addition, this estimate does not account for potential acclimation/adaptation in COTS gametes and developmental stages that may reduce effects of OA (see above). Although this figure is high, it must be interpreted in the context of other environmental parameters known to influence population development in this species. For example, a small increase in food availability for COTS larvae caused by terrestrial runoff can cause an 8-fold increase in larval survivorship [14]. The same study also showed that higher food availability can increase larval size whereas OA had no detectable effect on larval size in the present study. Together, these studies indicate that while OA may negatively affect population growth of this coral predator, improved understanding of the drivers of these outbreaks and potential management strategies are essential to control future outbreaks across the Indo-Pacific region. In addition, it is unlikely that reductions in COTS population size would take effect before corals themselves are impacted by OA or increased sea surface temperature [8], [9].

## Methods

### Ethics statement

All experiments were conducted in accordance with Australian laws and specimens collections were approved by the Great Barrier Reef Martine Park Authority (Permit No. G12/35236.1).

All experiments were conducted at 28°C which represents seawater temperatures in the source area of the *A. planci* (Green Island, Northern Great Barrier Reef, 16°46.5′S, 145°59.3′E) around spawning time (∼November to January).

### Fertilisation assay

Ovaries and testes were dissected from the base of the animals' arms. Testes spontaneously released sperm which was collected dry. Post-vitellogenic oozytes were obtained by treating dissected ovaries in 10^−5^ M 1-methyladenine in FSW [47].To represent a population response, fertilisation assays were conducted with the combined sperm from four males and oocytes from five females.

Experiments were conducted in 20 ml vials containing filtered (5 µm) natural sea water (salinity: 35.5) at the respective treatment pH. Dry sperm (0.1 ml) was diluted in 10.9 ml treatment water in each of 10 replicate vials for each *p*CO_2_ treatment to achieve an approximate sperm concentration of 10^7^–10^8^ sperm ml^−1^. A 1 ml sample was taken from each vial for sperm counts in a haemocytometer. We then diluted 1 ml of each replicate with 9 ml of water in six serial dilutions. To accurately fit statistical models, we calculated the sperm concentration in each vial as the measured concentration of the corresponding undiluted replicate multiplied by the dilution factor. We added 1 ml of oocyte suspension to each vial to achieve a final volume of 10 ml, and a concentration of 100 oocytes ml^−1^. After 10 minutes 2 ml were sampled from each vial, placed in an Eppendorf® tube and development stopped with 1 drop of 7% formalin. 50 eggs in each replicate were checked, the number of eggs with fertilization envelopes amongst those counted, and the percentage of fertilization calculated. Ambient pH_NBS_ (8.1), pH_NBS_ 7.9 and 7.6 were used as treatments representing present day and future (2100 to 2300) pH and *p*CO_2_ concentrations under different scenarios [48]. pH was measured using a temperature-corrected pH meter (OAKTON, USA; pH probe: EUTECH, USA), and controlled using a Tris standard (Dixon, Batch 5). Treatment sea water was prepared by slowly bubbling pure CO_2_ through a 10 l container of filtered seawater until the nominal target pH was reached. Stability of the pH was measured over the following 15 min, and for each treatment samples for alkalinity and dissolved organic carbon (DIC) analysis (250) were fixed with 125 µl of saturated mercuric chloride. Alkalinity/DIC Samples were analysed at 24°C using a VINDTA 3C titrator (Marianda, Germany). Calibration was conducted using Certified Reference seawaters (A. G. Dickson, Scripps Institute of Oceanography, Dixon, Batch 106).

We measured the pH of the coelomic fluid around the gonads of five *A. planci* individuals by carefully removing fluid around the gonads using a syringe. pH was measured directly after fluid removal with a pH probe as described above.

### Sperm velocity and motility, and pH of the coelomic fluid

Sperm point-to-point velocity (Velocity Curvi-Linear) and percentage of motile sperm (referred to as motility) were measured from 6 male *A. planci*, using similar techniques as were previously used for echinoids [20]. Sperm from each individual was kept at 28°C and assays conducted within 30 min. Control and treatment water was prepared in the same way as for the fertilisation assay. For each dilution, 2 µl of dry sperm were diluted with 4 ml of treatment water in a separate 2 ml scintillation vial.

Sperm dilutions were taken up in 0.3 mm capillary slides for microscopy. All slides were thoroughly washed and rinsed 3 times and coated in bovine serum albumin (1%) to avoid sperm sticking to the slides. We used a Zeis axioscope with 200X magnification for microscopic filming. The camera (Pixilink PL-B623) was set to take 25 frames per second over a two second period. Focus was set mid-plane to minimize wall effects on swimming speeds. From each slide at least 5 videos were taken to film a minimum of 200 sperm. Three slides from independent sperm dilutions were taken for each male x treatment combination.

For velocity analysis, we used ImageJ with the CASA (computer assisted sperm analysis) plugin. Motility was scored visually from the first 5 digital frames of each video counting all mobile and immobile sperm.

### Larval development

Larval development experiments were conducted in a flowthrough seawater system (∼180 L h^−1^) with UV sterilised and filtered (1 µm) water. The rearing containers were 100 ml plastic jars with a window that maintained a constant volume of 30 ml. A 45 µm mesh screen was glued in front of the widow to retain larvae. Experimental pH was regulated by injection of CO_2_ into the seawater reservoirs using an automatic CO_2_ injection system. The pH in sections of the system was regulated according to water chemistry conditions in the rearing containers with two pH controllers (Tunze), set at pH_NBS_, 7.6 and pH_NBS_ 7.8 with a third section allowed to track ambient pH.

Eggs from 4 females were pooled and placed in each rearing containers (N = 4) and allowed to acclimatise for a 15 min. Sperm of three males were pooled and added to achieve a sperm to egg ratio of 200∶1; 5×10^3^ sperm ml^−1^. The flow of seawater was turned off for 10 min to allow fertilisation. From day three larvae were fed three times a day with 1–15×10^6^ cells of *Proteomonas sulcata* for each container. At day 10 the larvae were harvested. The first 30 larvae were haphazardly sampled and scored for normal development. Normally developed larvae were photographed and their total length along the midline from posterior to the anterior, their maximum width and the length of the stomach from the end of the oesophageal tube to the beginning of the hind gut was measured.

For settlement experiments, larvae produced from the same males and females were reared in 300 l aerated containers (∼5 larvae ml^−1^). Larvae were fed daily with *P. sulcata* at approximately 10^5^ cells ml^−1^.

### Larval settlement

Crustose coralline red algae (CCA) of the species *Hydrolithon onkodes* were collected from Davies Reef (18°49.2′S, 147°37.9′E) in August 2012. That species was chosen because it was identified as a ‘refuge substratum’ for juvenile *A. planci* [49]; although one deep water species can induce higher settlement rates [28]. CCA chips (∼1 cm diameter) were embedded in non-toxic under water glue (Mr. Sticky's®, Fair Oaks, CA) to avoid open carbonate surfaces and glued to PVC slides. We also prepared slides only with the glue to test for potential toxic effects. Slides were mounted in custom perspex holders which were held in place on aquarium walls using magnets. The aquarium system used was similar to the system described for larval experiments. However, pH and temperature were monitored continuously (30 sec sampling rate) with ISFET type pH probes (Endress Hauser CPS-471D). Fresh filtered seawater (0.4 µm) was added to the system at a flow rate of 1 l min^−1^ for a 200% replacement rate per day. Flow rates in each experimental tank were 12 l min^−1^. In addition to a present day (pH_NBS_ 8.1 target), medium (future pH_NBS_ 8.0 target) and low (future pH_NBS_ 7.9 target) acidification treatments, this experiment included a pre-industrial treatment (past pH_NBS_ 8.25 target). Acidified treatments were achieved by bubbling CO_2_ into sump tanks with solenoid valves (SMC pneumatics) and pH setpoints, while the pre-industrial treatment was achieved by passing a stream of air through 2 sodalime canisters and mixing the low CO_2_ scrubbed air with the incoming seawater in a counter current exchange tower prior to flowing into each experimental tank. Temperatures were controlled with a heater chiller unit (EvoHeat DHP40) creating a recirculating water bath for temperature control. Seasonal temperature profiles were maintained with an average of 26.1°C±0.2 SD (Max = 27.9°C & Min = 24.0°C).

Settlement assays we conducted in 6 well tissue culture dishes (TCD). Each well contained 10 ml of FSW of the respective treatment. CCA were removed from the slide holders and carefully placed into the wells. We added 20–30 competent larvae to each well. Competent COTS larvae are late stage brachiolaria with a well-developed rudiment. Results from some wells which had a lower count of competent larvae at the end of the experiments were omitted from the analysis. The first experiment contained CCA pre-incubated in one of the 4 *p*CO_2_ treatments for 85 d and we ran 18 replicates for each treatment. In addition to the CCA we used biofilms grown on the glue for the same amount of time as additional settlement substratum. Six replicates were employed for each treatment.

To distinguish effects caused by changes in cues the CCA and biofilms growing under different *p*CO_2_ concentrations from those potentially caused by the differences in seawater chemistry, a second type of settlement experiment was conducted. In this experiment, all CCA (N = 12) used were grown under “present day” conditions. During the settlement period, these CCA and the larvae were inserted into the wells together with water from the 4 different treatments. Both types of experiments were run in parallel with larvae of the same age and maturity. In order to minimise re-equilibration of the CO_2_ in treatment water to present day conditions, 6 well plates were closed with a lid, and floated individually in closed 400 ml plastic containers containing 200 ml of treatment water.

### Statistics

Fertilisation rates at different sperm concentration were modelled using a two-parameter logistic model (asymptote fixed to 100), with log-transformed sperm concentrations. Standard errors for the predicted curves where estimated using the delta method, a method used in statistics to derive approximate probability distributions. These standard errors were used to calculate confidence intervals.

A total of 38,121 sperm were analysed for velocity and motility. We used the average velocity per replicate within each slide for statistical analysis. Mixed model Analyses of Variance (ANOVA) was used to analyse both sperm velocity and motility. pH/*p*CO_2_ treatments and Males were treated as fixed factors, whereas replicate dilutions (slide) were treated as a random/nested factor. Sperm velocity was log transformed and motility arcsine-square root-transformed prior to analysis.

Data for development were analysed with a one-factor (% normal development, arcsine-square-root transformed) or mixed model (larval size, rearing container used as nested factor) ANOVA.

Data for larval settlement (% settlement of competent larvae) were arcsine-square root-transformed and subjected to two-factor ANOVA with Treatment (4 different pH/*p*CO_2_ levels) and CCA vs biofilm as fixed factors. Type III sums of squares were used for this ANOVA because removal of some replicates with low numbers of competent larvae (see above) and unequal sample size between CCA and biofilm-controls lead to an unbalanced design. One way ANOVA (also Type III) was used to for the water-control experiment. Tukey-Kramer tests were choses as post-hoc tests for all ANOVA if indicated. Transformed data for all ANOVA conformed to normality and equal variance assumptions.

To compare the effect size of all individual parameters, the change in response was expressed as a precent change compared to control (present day) conditions. Based on the standard deviations of the control and respective treatment, we calculated a Bayesian 95% confidence interval for the reduction using Markov Chain Monte Carlo sampling. After a burn-in period of 2000 steps, 5000 steps were sampled and three parallel chains run.

Statistical analyses were conducted in R[50], and mixed model ANOVAs conducted in NCSS [51].
