# Correction: Impacts of Ocean Acidification on Early Life-History Stages and Settlement of the Coral-Eating Sea Star *Acanthaster planci*

**DOI:** 10.1371/annotation/b03dc5d7-0cfd-4182-b39d-fb9299275d5c

**Published:** 2014-01-07

**Authors:** Sven Uthicke, Danilo Pecorino, Rebecca Albright, Andrew Peter Negri, Neal Cantin, Michelle Liddy, Symon Dworjanyn, Pamela Kamya, Maria Byrne, Miles Lamare

Two funding organizations are incorrectly omitted from the Funding Statement. The Funding Statement should read: "Funding was provided by the Australian Institute of Marine Science, the Australian Government’s National Environmental Research Program and an Australian Research Council Grant. The funders had no role in study design, data collection and analysis, decision to publish, or preparation of the manuscript.”

